# Legionnaires’ disease after using an industrial pressure test pump: a case report

**DOI:** 10.1186/1752-1947-8-31

**Published:** 2014-01-27

**Authors:** Sjoerd M Euser, Bas Boogmans, Petra Brandsema, Mieke Wouters, Jeroen W Den Boer

**Affiliations:** 1 Regional Public Health Laboratory Kennemerland, Boerhaavelaan 26, 2035, RC Haarlem, The Netherlands; 2 Municipal Health Service, Veiligheids- en Gezondheidsregio Gelderland Midden, Eusebiusbuitensingel 43, 6828, HZ Arnhem, The Netherlands; 3 National Institute for Public Health and the Environment, Antonie van Leeuwenhoeklaan 9, 3721, MA Bilthoven, The Netherlands

**Keywords:** Diagnostic testing, Industrial pump, *Legionella*, Legionnaires’ disease, Source investigation

## Abstract

**Introduction:**

Legionnaires’ disease is an acute pneumonia caused by inhalation or aspiration of aerosols contaminated with *Legionella* bacteria. The majority (>90%) of Legionnaires’ disease cases are caused by the species *Legionella pneumophila*, and about 85% more specifically by *L. pneumophila* serogroup 1 that can be detected by a fast and easy to perform urinary antigen test. Previously reported sources of infection include cooling towers, plumbing systems of hospitals, and whirlpool spas, but for the majority of cases of Legionnaires’ disease the source of infection remains unknown.

**Case presentation:**

A 52-year-old Caucasian man was admitted to a Dutch hospital with pneumonia, where a culture of the available bronchial lavage was found positive for *L. pneumophila* serogroup 3, confirming the diagnosis of Legionnaires’ disease. An environmental investigation identified a manually operated pressure test pump at the metal processing company where he worked as the source of infection: the water sample from the pump contained 9·8×10^3^ colony forming units/L *L. pneumophila*, and sequence-based typing showed the same sequence type (ST93) for both the clinical and environmental strains.

**Conclusion:**

This case shows that Legionnaires’ disease can be acquired by exposure to relatively rare sources that are not considered in regular control and prevention measures.

## Introduction

Legionnaires’ disease (LD) is an acute pneumonia caused by *Legionella* species, with the major route of transmission being inhalation of the bacterium that is disseminated into the air as an aerosol from either natural or human-made sources [1]. It was named after a point-source outbreak in a hotel that hosted the convention of the American Legion in 1976 [2,3]. The disease is characterized by an acute pneumonia, a low attack rate (0.1 to 5%) and an average incubation time of 2 to 10 days, although it may even exceed this period [1-4]. LD is thought to account for 2 to 15% of all community-acquired pneumonias, and proves fatal in about 6% of cases [1,5-7]. The majority (>90%) of LD cases are caused by the species *Legionella pneumophila*, and about 85% more specifically by *L. pneumophila* serogroup 1 [8,9].

## Case presentation

In February, 2012, a 52-year-old Caucasian man with no underlying disease (but a cigarette smoker) was admitted to the intensive care unit of a Dutch hospital, with high fever, shortness of breath, electrolyte imbalance, diarrhea, and neurological symptoms. Chest radiography showed areas of consolidation in both his lungs and confirmed the diagnosis of pneumonia. He was treated with intravenous ciprofloxacin (400mg daily), intravenous cefuroxime (6 days, 1000mg every 8 hours), and doxycycline oral (3 days, 200mg twice daily). Following negative results for both a *L. pneumophila* urinary antigen test, and a polymerase chain reaction (PCR) assay that targeted the 5S ribosomal deoxyribonucleic acid (DNA) gene performed on a sputum sample (DNA extraction by NucliSENS® easyMAG®, bioMérieux, Durham, USA), treatment with intravenous ciprofloxacin was stopped after 4 days [10]. Two days later, both PCR and culture of a bronchial lavage sample were found positive for *L. pneumophila* (serogroup 3), and the treatment with intravenous ciprofloxacin was continued for another 10 days. The patient left the intensive care unit after 13 days, recovered and left the hospital after 23 days.

**Figure 1:**
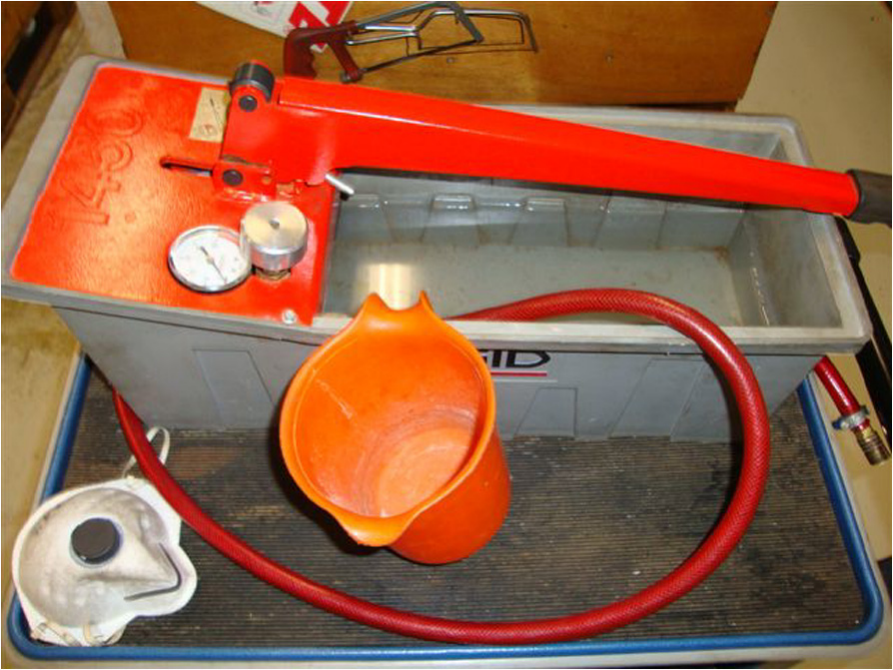
Pressure test pump.

In accordance with the National Legionella Outbreak Detection Programme that was installed in the Netherlands in 2002, an investigation was performed to find the source of infection [11]. During the source investigation, two potential sources were identified: (1) the house of the patient where he had used taps and shower, and (2) the metal processing company where he worked and was exposed to water-based cutting fluids, and a pressure test pump (Figure 1) that uses water to evaluate the quality of the produced industrial iron molds. With this manually operated pressure test pump, molds are tested for leakages by pushing water through the mold with increasing air pressure. When a leak is present, respirable water aerosols are sprayed around by the pump, and the pressure drops. Samples were taken from the patient’s home (taps and shower), from the cutting-fluids and water reservoir of the pump at the company. All nine samples from his home were negative, but one of the five samples from the company was positive for *L. pneumophila.* The sample from the water reservoir of the pressure test pump contained 9·8×10^3^ colony forming units/L. Both *L. pneumophila* serogroup 1 and serogroup 3 were isolated from this sample. Sequence-based typing (SBT) showed the same sequence type (ST93) for both the clinical and environmental *L. pneumophila* serogroup 3 strains [12]. This sequence type was previously reported for only 29 clinical and 15 environmental isolates according to the European Working Group for Legionella Infections SBT database which contains sequence types of over 6600 *L. pneumophila* strains [13]. The high pressure pump was dismantled and thoroughly cleaned and rinsed several times, and control measures and changes in the working procedure were implemented. Employees are now required to wear filtering facepiece 2 respirator masks when operating the pump and the water reservoir is emptied and dried after every use. No other cases of LD related to this company were reported among the other employees.

## Conclusions

The majority of LD cases (approximately 79%) are caused by *L. pneumophila* serogroup 1, although this may well be influenced by the common use of diagnostic tests (urinary antigen tests) that are sensitive for *L. pneumophila* serogroup 1, but have difficulties in detecting other serogroups [1]. The case reported here demonstrates the importance of using additional diagnostic methods (culture), besides the fast and easy to perform urinary antigen tests, to obtain a more accurate diagnosis. Furthermore, this case shows that LD can be acquired by exposure to relatively rare (not previously reported) sources that are not considered in regular control and prevention measures that are applied to well-known potential sources of LD like cooling towers and the plumbing systems of hospitals.

## Consent

Written informed consent was obtained from the patient for publication of this case report and any accompanying images. A copy of the written consent is available for review by the Editor-in-Chief of this journal.

## Abbreviations

LD: Legionnaires’ disease; PCR: Polymerase chain reaction; SBT: Sequence-based typing.

## Competing interests

The authors declare that they have no competing interests.

## Authors’ contributions

BB and MW contributed to the data collection, analysis and interpretation of data, and preparation of the manuscript. PB contributed to the analysis and interpretation of data, and preparation of the manuscript. JWDB contributed to the data collection, analysis and interpretation of data, and preparation of the manuscript. SME contributed to the data collection, analysis and interpretation of data, and drafted the manuscript. All authors read and approved the final manuscript.
